# The benefit of deep genomic testing for asymptomatic high-risk individuals undergoing surveillance for pancreatic adenocarcinoma: European registry for hereditary pancreatic diseases (EUROPAC-PLUS)

**DOI:** 10.1016/j.neo.2026.101316

**Published:** 2026-05-22

**Authors:** Phillip J. Hopley, Annabelle Boughey, Katie Bullock, William Greenhalf, Christopher M. Halloran

**Affiliations:** a Department of Molecular and Clinical Cancer Medicine, William Henry Duncan Building, University of Liverpool, West Derby Street, Liverpool, England, UK; b Liverpool University Hospitals NHS Foundation Trust, Mount Vernon Street, Liverpool, England, UK

**Keywords:** Pancreatic ductal adenocarcinoma, High risk individuals, Surveillance, Germline genetic testing

## Abstract

**Introduction and Aims:**

Surveillance is recommended for High-Risk Individuals (HRI) who are predisposed to pancreatic ductal adenocarcinoma (PDAC), because of familial (i.e., genetic) predisposition. All individuals under consideration for such surveillance in the UK, are registered and risk scored by the European Registry for Hereditary Pancreatic Diseases (EUROPAC). We generated a family risk score (FR) to optimize pancreatic cancer surveillance, started using this in 2020 and have recently validated it. Our aim was to combine this score *with* germline DNA analysis from both candidates and affected family members to form improved and distinct groups for surveillance.

**Methods:**

Thermo-Fischer™ Ion AmpliSeq Custom Targeted NGS Panel of 43 genes, was undertaken on participant samples. Genes selected were based on known associations with germline cancer predisposition or overlapping gene ontology with cancer predisposition genes. Variants discovered were classified according to their pathogenicity.

**Results:**

Between 1st April 2022 and 1st December 2025, 271 individuals samples underwent analysis. 37 participants showed 38 mutations with sufficient complexity to require both check analysis and additional discussion at the Geno-Phenotype Multi-Disciplinary Team. In 26 (26/271; 10%) of these, a mutation was detected which either directly affected surveillance *or* affected outcomes. All gene variants were subjected to oversight by authors who can interpret both their genetic and clinical importance. Of note our deep genomic search discovered nine variants which are currently unavailable on the NHS or National Comprehensive Cancer Network.

**Conclusions:**

Comprehensive genomic analysis of families at risk of developing PDAC, better identifies those individuals in need of surveillance.

## Highlights

• High Risk Individuals (HRI) should undergo risk-stratified surveillance for PDAC.
• Most HRI are ineligible for genetic testing, denying entrance into surveillance.
• Extended germline testing can find unknown pathogenic variants which reverses this.

## Introduction

Pancreatic ductal adenocarcinoma (PDAC) is on track to be the 2nd commonest cause of cancer death within this decade, with a 5-year survival globally of <10% [1]. Therefore, undertaking surveillance programs in High-Risk Individuals (HRI) who are predisposed to PDAC, should detect malignancy earlier and at earlier stage such that curative treatment is possible. Such directed surveillance is recommended for those at familial (i.e., genetic) predisposition of PDAC [[2], [3], [4]], namely those with a family history of PDAC who are not known to carry a germline pathogenic variant (FPC PV-), those with a family history of PDAC and with a germline pathogenic variant (FPC PV+), or those with hereditary pancreatitis (*PRSS1* pathogenic variants). Familial Pancreatic Cancer is defined as two first-degree relatives with PDAC (one of whom is the first degree relative of the proband) on the same side of the family or a family with three or more relatives with PDAC on the same side of the family. In the UK the National Institute for Health and Care Excellence (NICE) recommends that such HRI’s have annual assessment as part of a research program [5] that monitors outcomes and allows for contemporaneous research. All individuals under consideration for such surveillance in the UK, are registered and risk scored by the European Registry for Hereditary Pancreatic Diseases (EUROPAC) [6]. The Family Risk score (FR) is based on family history, the likelihood of carrying a pathogenic variant (PV) or conditional risk beyond coincidence and determines whether individuals should be offered pancreatic cancer surveillance. An FR score of >30, denotes suitability for surveillance [7]. Moreover, EUROPAC has recently published and validated this risk-stratification [8], in nearly 900 participants. These include 673 FPC PV-, 182 HPC PV+ and 38 with HP. Specifically, the FPC PV- group contains 100/673 (15%) who were eligible for genetic testing and in whom no PV was found. The remaining 573/673 (85%) were not eligible for genetic testing, by UK genetics criteria, at the time of their registration. Overall, 18 participants (2%) have been found to have lesions requiring resection or palliative care, which is comparable to UK bowel and lung surveillance pathways. 78% of these lesions were pre-malignant or early-stage malignancy (≤Stage II) at diagnosis.

Although a breakthrough publication, it does raise some questions about our surveillance strategy which is based a number of assumptions: (a) That patients with a germline PV which confers risk for pancreatic cancer *do not* have conditional familial risk (FR); (b) That patients with an adequate family risk (i.e., a FR >30) could not be better stratified for risk by genetic testing and (c) That patients with a low family risk (i.e., a FR <30) who are not surveyed, have limited risk, and could not be better stratified for risk by genetic testing. The aim was to combine our validated FR score *with* germline DNA analysis from both candidates and affected individuals within their family (where available). As identification of a pathogenic variant automatically at least doubles an individual’s risk score (see calculation [7]), with potential consequences for the eligibility of other family members for surveillance [9], this will form new distinct groups for surveillance, with an algorithm change for the perceived highest risk.

## Methods

### Calculation of Risk Score (RS)

**Fig. 1:**
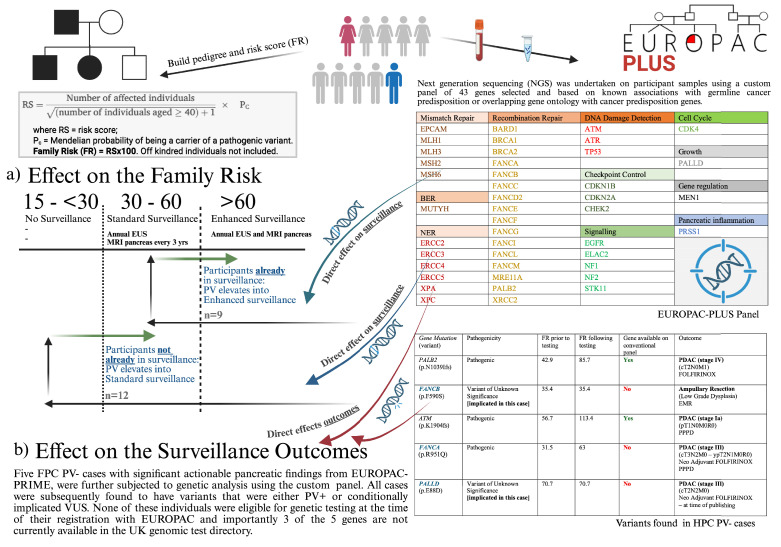
This shows the two main aspects of this work. a) Calculation and effect of the Family Risk (FR). Three groups are shown: FR 15-30 which signified a population who do not have a risk high enough to be eligible for surveillance; FR 30-60 which signified a population who have a risk high enough to be eligible for STANDARD surveillance and FR >60 which signified a population who have a risk high enough to be eligible for ENHANCED surveillance. The blue arrows signify where a PV is found which moves 12 participants from non-surveillance to STANDARD surveillance and nine participants from STANDARD to ENHANCED surveillance. b) Direct effect on outcomes of EUROPAC-PRIME. The 5 FPC (PV-) individuals when tested were all found to contain either a PV or a conditionally implicated VUS (red arrow). *This figure was Created in*https://BioRender.com*. We hold a publication license from BIORENDER for this*.

RS will be calculated as [7]:

$$RS=\frac{\text{Number}\text{of}\text{affected}\text{individuals}\;}{\sqrt{\left(\text{number}\text{of}\text{individuals}\text{aged}\;\geq 40\;\right)+1}}\times P_{C}$$

where RS = risk score; P_c_ = Mendelian probability of being a carrier of a pathogenic variant. Family Risk (FR) = RSx100. Off kindred individuals not included.

(score of ≥30 signifies the surveillance threshold)^.^Fig. 1.

### Next generation sequencing (NGS) EUROPAC-PLUS panel

Thermo-Fischer™ Ion AmpliSeq Custom Targeted NGS Panel of 43 genes, (Ion 530 chips on the GeneStudio S5 sequencer) was undertaken on participant samples, following informed consent. Genes selected were based on known associations with germline cancer predisposition or overlapping gene ontology with cancer predisposition genes. All tested individuals were scrutinised in a geno-phenotype Multi-Disciplinary Team - MDT (CH, WG, AB and PH), where genetic variants were evaluated against data in ClinVar (NIH), in the context of the individual and with clinical relevance prior to being classified as: Benign, Likely Benign, Variant of Unknown Significance (VUS), Likely Pathogenic or Pathogenic. Likely Pathogenic / Pathogenic variants were considered to increase an individual’s risk, FR doubled (defined as pathogenic variants; PV), while Likely Benign / Benign variants did not affect the FR score (defined as non-pathogenic variants). The finding of a VUS had a conditional outcome, depending upon the descision from the MDT or whether an actionable finding occurred in that individual during surveillance. Where individuals were found to carry more than one variant, as outlined above, the most significant variant was recorded Fig. 1.

### Outcomes

Effect upon Surveillance: Whether the finding of a PV elevates a participant from standard to enhanced surveillance, from no surveillance to standard surveillance or has no effect. Effect upon Outcomes: We validated germline mutations found in individuals who develop PDAC in surveillance where one has not previously been identified in the family [8] Fig. 1.

## Results

Between 1st April 2022 and 1st December 2025, 295 individuals with samples were identified who may benefit from germline testing, 271 underwent analysis. 231/271 (85%) of these participants were found to have non-pathological variants, in whom surveillance or outcomes was not affected: 78 with benign mutations, 22 with likely benign mutations, 115 with VUS and 16 with conflicting outcome data where it was not possible to come to a safe conclusion. A further 14 (5%) were found to have no mutations, in whom surveillance was also not affected. 37 participants showed 38 mutations with sufficient complexity to require check analysis and additional discussion at the Geno-Phenotype MDT. In 26 (26/271; 10%) of these, a PV was detected which either directly affected surveillance (n = 21), Table 1 *or* affected outcomes (n = 5) from surveillance, Fig.1.

**Table 1:** Results of EUROPAC-PLUS genetic testing.

Individual	*Gene Mutation* (variant)	Pathogenicity	FR prior to testing	FR following testing	Outcome on Surveillance	Refer to clinical genetics	Outcome
**1.**	***ATM*** (p.K1904fs)	Pathogenic	56.7	113.4	Increase	Yes	**Enhanced**
**2.**	***ATM*** (p.Q1852X)	Pathogenic	56.7	113.4	Increase	Yes	**Enhanced**
**3.**	FANCG* (p.W572X)	Pathogenic	55.9	111.8	Increase	No*	**Enhanced**
**4.**	***PALB2*** (p.N1039Ifs)	Pathogenic	51.45	102.9	Increase	Yes	**Enhanced**
**5.**	FANCA* (p.D539fs)	Pathogenic	40.8	81.6	Increase	No*	**Enhanced**
**6.**	***BRCA1*** (p.E23fs)	Pathogenic	40.8	81.6	Increase	Yes	**Enhanced**
	***BRCA2*** (p.S1982fs)	Pathogenic					
**7.**	***BRCA2*** (p.L2357fs)	Pathogenic	35.4	70.8	Increase	Yes	**Enhanced**
**8.**	ERCC2* (p.R112C)	Likely Pathogenic	33.3	66.6	Increase	No*	**Enhanced**
**9.**	***CDKN2A*** (p.I49S)	Likely Pathogenic	30.2	60.3	Increase	Yes	**Enhanced**
**10.**	***CDKN2A*** (p.M53I)	Pathogenic	24.2	48.4	Increase	Yes	**Standard**
**11.**	***CDKN2A*** (p.M53I)	Pathogenic	22.4	44.8	Increase	Yes	**Standard**
**12.**	FANCM* (p.R1931X)	Pathogenic	20	40	Increase	No*	**Standard**
**13.**	***ATM*** (p.S2017Kfs)	Pathogenic	18.6	37.2	Increase	Yes	**Standard**
**14.**	ERCC-2** (p.A717G) (p.L461V)	Pathogenic	27.7	55.5	Increase	No*	**Standard**
**15.**	***ATM*** (p.K468fs)	Pathogenic	18.3	36.6	Increase	Yes	**Standard**
**16.**	***BRCA2*** (p.L2357fs)	Pathogenic	26.7	53.4	Increase	Yes	**Standard**
**17.**	***BRCA2*** (p.L2357fs)	Pathogenic	26.7	53.4	Increase	Yes	**Standard**
**18.**	***PALB2*** (p.S168X)	Pathogenic	26.7	53.4	Increase	Yes	**Standard**
**19.**	***PALB2*** (p.S168X)	Pathogenic	26.7	53.4	Increase	Yes	**Standard**
**20.**	ELAC2*^§^(p.R781H)	Likely Pathogenic	27.9	55.8	Increase	No*	**Standard**
**21.**	ELAC2*^§^(p.R781H)	Likely Pathogenic	22.9	45.8	Increase	No*	**Standard**

Table 1. This is split into two sections, individuals who were elevated from STANDARD surveillance to ENHANCED surveillance, those who were elevated from no surveillance to STANDARD surveillance. *GENES* are *italicised,* the genetic variant in brackets. The participant with two PV’s, had both a *BRCA1 and a BRCA2* mutation, as both were equally pathogenic only one was counted. Overall, there were nine PV found that are not included on the UK genomics test directory and therefore are unavailable on the NHS (*). The *ERCC2* (**) gene is commonly found with 2 PV’s, also found here, as it is not clear whether both are required for pathogenicity both are reported. This gene is also not on the afore mentioned genomic panels. We have classified these mutations (^§^) as likely pathogenic following the geno-phenotype MDT, and the role of *ELAC2* will be discussed in a future paper.

### EUROPAC-PLUS found pathogenic variants which moved participants between surveillance groups

Table 1 demonstrates that in 9 participants (9/271, 3%), who were already in EUROPAC surveillance, the discovery of a PV increased their FR score such that they were now eligible for enhanced surveillance. Additionally, 12 participants (12/271, 4%), who were not in EUROPAC surveillance because their FR was <30, the discovery of a PV increased their FR score such that they were now eligible for standard surveillance.

### EUROPAC-PLUS did not influence surveillance in some participants

Of the 37 individuals who were discussed at the geno-phenotype MDT, it was concluded in 11 cases (eight with a FR<30, not in surveillance and three with an FR>30, in surveillance), that there was insufficient evidence to consider the genetic variant was pathogenic in that individuals context and as such their surveillance was not altered.

### *EUROPAC-PLUS used in post-hoc analysis of FPC PV- outcomes for EUROPAC PRIME* [8]

These latter cases were analysed from the EUROPAC-PRIME cohort, where Five FPC PV- cases with significant actionable pancreatic findings, were subjected to a posteriori EUROPAC-PLUS NGS and found to have variants that were either PV+ or conditionally implicated VUS, Fig. 1.

## Discussion

We have demonstrated that by utilising Family risk *with* germline DNA from both candidates and affected individuals within their family (where available) that an algorithm change for those perceived to be at the highest risk, can be achieved. Although most individuals will not have a pathogenic mutation, with a few having no mutations found even in expanded panels, around 10% will have either a PV or conditional VUS which can directly affect surveillance or outcomes from surveillance. The obvious and immediate benefit of such a system is when participants can be made eligible for standard surveillance or be elevated to higher intensity of surveillance (enhanced) as their risk scores (FR) increases. This then has ramifications to the other members of the family if they also carry the same or similar PV’s, whilst sparing those who do not carry such mutations, from rounds of potentially un-useful surveillance.

All gene variants should be subjected to intense scrutiny, by experts who can interpret both their genetic and clinical importance. EUROPAC has operated a geno-phenotype MDT since 2019 to specifically review such findings. This work excluded 11 variants as we could not be certain of their consequence. Criteria for germline genetic testing under the UK Genomic Test Directory are restrictive [10], when compared to the National Comprehensive Cancer Network [11], although both have limited genetic depth and utilisation even in advanced health care economies [12]. Both in this work and that of Hopley et al [7], the custom Thermo-Fischer™ EUROPAC-PLUS panel has found nine PV’s within genes, none of which are available currently on the NHS or NCCN which not only change suitability for surveillance but also the risk for developing PDAC or its precursor lesions. This emphasises the need for expansion of genetic panels to thoroughly identify all valuable variants rather than just those commonly available.

## Conclusions

We have shown that providing comprehensive genomic analysis to families at risk of developing PDAC, better identifies those individuals in need of surveillance and those who may develop subsequent lesions but also reduces surveillance where it will be ineffective.

## Declaration of generative AI use

Generative AI was not used in any way to perform this research or write this manuscript

## Ethical approval

This study was conducted in accordance with the ethical standards in the Declaration of Helsinki. Ethical approval was granted by the Yorkshire and Humber Research Ethics Committee (Ref: 19/YH/0250).

## Funding

This work was supported through an early detection grant awarded to Prof C M Halloran.

## Data availability

Data are available from the corresponding author upon reasonable request.

## CRediT authorship contribution statement

**Phillip J. Hopley:** Data curation, Formal analysis, Writing – original draft, Writing – review & editing. **Annabelle Boughey:** Data curation, Project administration, Software, Validation, Writing – review & editing. **Katie Bullock:** Resources, Validation, Writing – review & editing. **William Greenhalf:** Conceptualization, Data curation, Validation, Writing – original draft, Writing – review & editing. **Christopher M. Halloran:** Conceptualization, Formal analysis, Funding acquisition, Supervision, Validation, Writing – original draft, Writing – review & editing.

## Declaration of competing interest

The authors declare the following financial interests/personal relationships which may be considered as potential competing interests: CM Halloran reports equipment, drugs, or supplies was provided by Cheshire and Merseyside Cancer Alliance. The authors declare the following financial interests/personal relationships which may be considered as potential competing interests: CMH has grants from NHS England, Cancer Research UK, Pancreatic Cancer UK and Liverpool University Hospitals NHS Foundation Trust. CMH is a scientific advisor for Galleri™. CMH and WG are named as inventors on GB patent GBGB1806002⋅0; PCT/GB2019/050998, submitted by the University of Liverpool, that covers the measurement of adiponectin and IL-1Ra as a biomarker for early detection of pancreatic cancer. All remaining authors have declared no conflicts of interest. If there are other authors, they declare that they have no known competing financial interests or personal relationships that could have appeared to influence the work reported in this paper.
